# Functional and Aesthetic Treatment of Patients With Tooth Structure Anomalies: A Narrative Review

**DOI:** 10.7759/cureus.88878

**Published:** 2025-07-28

**Authors:** Ann Katrin Reuter, Vicky Ehlers

**Affiliations:** 1 Department of Periodontology and Operative Dentistry, University Medical Center of the Johannes Gutenberg University Mainz, Mainz, DEU

**Keywords:** aesthetic treatment, amelogenesis imperfecta (agi), dentinogenesis imperfecta (dgi), functional treatment, tooth structure anomalies

## Abstract

Amelogenesis imperfecta and dentinogenesis imperfecta are two rare inherited disorders of tooth structure. Because of their rarity, there are no treatment guidelines so far. Therefore, it is difficult for dentists to decide which individual treatment is best for the patient, as they have to consider the psychosocial impact, function, aesthetics, and structural changes to the tooth structure. This paper attempts to find out what treatment options have been used worldwide and which have worked best through a literature review.

## Introduction and background

Amelogenesis imperfecta and dentinogenesis imperfecta are rare inherited dental diseases characterised by abnormalities in tooth structure. In amelogenesis imperfecta, the enamel is thin or absent, while in dentinogenesis imperfecta, the dentin is affected, often resulting in discoloured, brittle teeth. Both conditions significantly impact dental function and aesthetic appearance, with potentially severe psychological and social consequences, particularly for children and adolescents [1,2].

According to Wright JT et al., amelogenesis imperfecta is a group of hereditary disorders affecting the development and mineralisation of the tooth enamel [3]. Several genes associated with amelogenesis imperfecta have been identified, including amelogenin (AMELX), enamelin (ENAM), matrixmetalloproteinase 20 (MMP20), protein coding: family with sequence similarity 83 member H (FAM83H), WD repeat-containing protein 72 (WDR72), ameloblastin (AMBN), homeobox-protein DLX-3 (DLX3), and kallikrein-related peptidase 4 (KLK4) [4,5]. However, amelogenesis imperfecta occurs in approximately one in 7,000 to 14,000 individuals [4] and dentinogenesis imperfecta in one in 6,000 to 8,000 [6,7]. Both amelogenesis imperfecta and dentinogenesis imperfecta are classified into different types. It is known that dentinogenesis imperfecta type II and dentinogenesis imperfecta type III are associated with a gene mutation of the dentin sialophosphoprotein (DSPP), which is highly expressed in odontoblasts and is located on chromosome 4q21.3 [8]. However, the management of these conditions presents a considerable clinical challenge. Treatment is often complex and time-consuming and may cause an economic burden on the patient's family. A tailored, multidisciplinary approach is required, which may include restorative procedures, orthodontics, and prosthetic rehabilitation, taking into account the patient's age, stage of development, and aesthetic and functional needs [9].

This review article evaluates current case reports and studies to identify effective treatment strategies for patients affected by amelogenesis imperfecta or dentinogenesis imperfecta.

## Review

Materials and methods

The basis of this analysis was a PubMed search up to March 2, 2024 using the MESH terms “(structural anomalies AND dental) AND (rehabilitation OR functional) AND aesthetic”, “(Amelogenesis Imperfecta) AND (oral AND rehabilitation) AND functional AND aesthetic” and “(Dentinogenesis Imperfecta) AND (oral AND rehabilitation) AND functional AND aesthetic”. This search identified 184 articles. After deletion of duplicate or off-topic publications, 53 case reports and two study reports remained.

The analysis involved a data collection process based on the following PICO question: Patient/Population: patients diagnosed with amelogenesis imperfecta or dentinogenesis imperfecta; Intervention: functional and/or aesthetic treatment; Controlled Intervention: no controlled intervention was applied; Outcome: how were the patients with amelogenesis imperfecta or dentinogenesis imperfecta treated?

**Figure 1:**
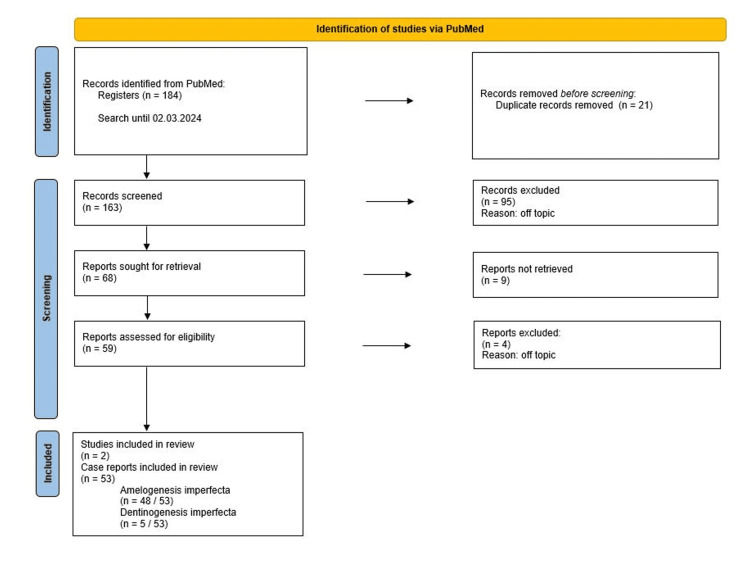
Preferred Reporting Items for Systematic Reviews and Meta-Analysis (PRISMA) flowchart

The selection procedure for the studies was carried out using a Preferred Reporting Items for Systematic Reviews and Meta-Analysis (PRISMA) flowchart (Figure 1) [10].

Inclusion criteria were conditions with amelogenesis imperfecta or dentinogenesis imperfecta with aesthetic and/or functional treatment. Exclusion criteria were duplicate records, off-topic records, and records that were not retrieved. The results were summarised in tables and checked for clinical relevance.

Results

Geographical Distribution

The geographical distribution of the case reports and study reports is shown in Table 1. These reports were published in different countries, such as India, Turkey, Brazil, France, China, Iran, and the USA, where most of the cases occurred. For diagnostic criteria, amelogenesis imperfecta and dentinogenesis imperfecta were classified using clinical criteria or genetic testing.

**Table 1:** Countries in which the case reports and the study reports have been described

Reports	Country	Quantity
Amelogenesis imperfecta case reports (n = 48)	India	n = 12
	Turkey	n = 8
	Brazil	n = 7
	France	n = 4
	China	n = 3
	Iran	n = 3
	USA	n = 3
	Switzerland	n = 2
	Germany	n = 1
	Greece	n = 1
	Pakistan	n = 1
	Saudi Arabia	n = 1
	Tunesia	n = 1
	UK	n = 1
Dentinogenesis imperfecta case reports (n = 5)	USA	n = 2
	France	n = 1
	India	n = 1
	Saudi Arabia	n = 1
Amelogenesis imperfecta study reports (n = 2)	Sweden	n = 1
	Taiwan	n = 1

Amelogenesis Imperfecta

**Figure 2:**
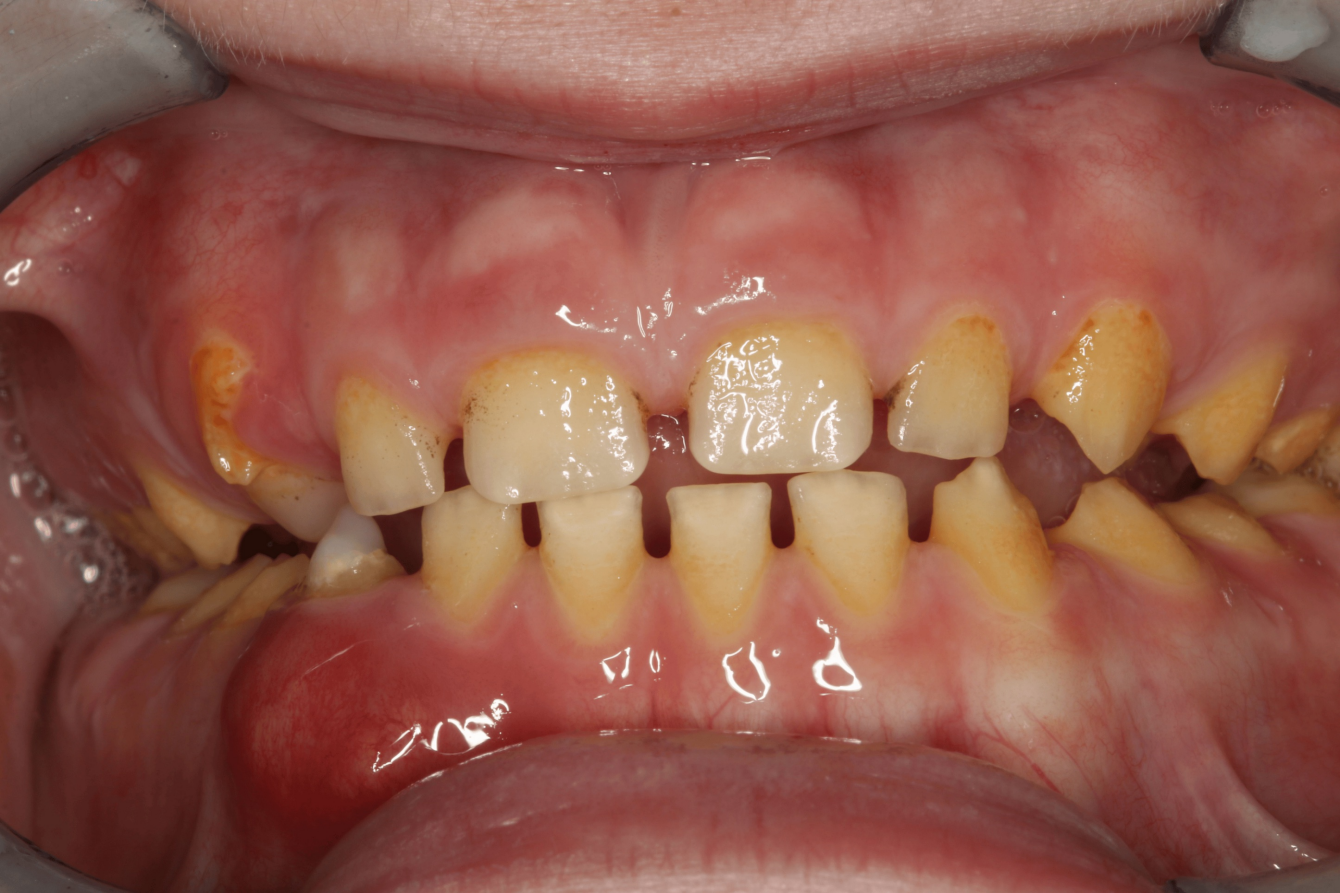
Male patient with amelogenesis imperfecta

Figure 2 shows an 11-year-old boy with amelogenesis imperfecta.

Definition and Classification

Amelogenesis imperfecta is a rare disease with an estimated prevalence of one in 14,000 people [4]. According to Witkop's classification, there are four main types [1]: Type I (hypoplastic): enamel with reduced thickness, often accompanied by large superficial defects [11-14]; Type II (hypomaturation): reduced enamel strength, often associated with a clinically opaque or mottled appearance [1,13-15]; Type III (hypocalcified): softer, more fragile enamel prone to abrasion [1,14-15]; Type IV: mixed form of hypomaturation and hypoplastic combined with taurodontism [1,16-17].

Clinical Criteria

Characteristic findings of amelogenesis imperfecta are enamel discolouration, attrition, and increased sensitivity [1,14-15]. The aesthetic and functional limitations have a negative impact on chewing ability and quality of life. Children and adolescents may be affected by psychological consequences [18].

Treatments

Analysis of the 48 case reports shows that treatments should be assessed on an individual basis. The most common treatments included restorative rehabilitation, for which composite fillings, veneers, and crowns were used to restore the patients' aesthetics and functionality [8,19-58]. In prosthetic treatment, implants or dentures were used in cases of severe tooth loss [21,53,59]. Surgical treatments such as gingivoplasty and orthodontic surgery may also be required [21-22,24,28-29,33,35-36,40,42-43,47-48,52,57-58,60-61]. Long-term temporary crowns were used to compensate for altered vertical relationships and protect affected teeth [8,24-25,28,33,37,39-40,42,44,48,55,57,61-62].

Clinical Trials

Two clinical trials were found in this analysis (Table 2): The first study by Lindunger and Smedberg involved 15 patients with hypoplastic and hypomatous amelogenesis imperfecta who underwent restorative rehabilitation involving extensive coronal restorations. The success rate was 99.5%, with an average durability of 60 months. [63]. The second study by Chen et al. involved eight patients aged eight to 18 years who had a combination of steel crowns, composite veneers, and crowns. The results were positive, with perhaps 10 restorations showing failure [64]. 

**Table 2:** Study reports of amelogenesis imperfecta

Author, year	Lindunger and Smedberg, 2005 [63]	Chen et al., 2013 [64]
Country	Sweden	Taiwan
Study	Clinical trial	Clinical trial
Quantity	n = 15	n = 8
Patients age	14 – 37 years; median: 23 years	8 – 18 years; median: no specification
m / f	7 / 8	2 / 6
Hypoplastic	10	4
Hypocalcified	-	2
Hypomaturation	5	2
Number of teeth	n = 403	n = 67
Steel crown	-	n = 27
Amalgam	-	n = 4
Direct restoration	-	n = 21
Indirect restoration	n = 213; 121 porcelain-fused-to-metal crowns, 36 veneers, 28 all-ceramic crowns, 18 ceramic onlays, 10 gold crowns	n = 15; no indication of the number of laboratory-fabricated composite veneers and laboratory-fabricated composite or acrylic crowns
Range / mean age of the restoration	12 – 240 months; median: 60 months	38.53 months; median: no specification
Results	212 / 213 satisfactory to excellent	10 restorations à inacceptable

Dentinogenesis Imperfecta

**Figure 3:**
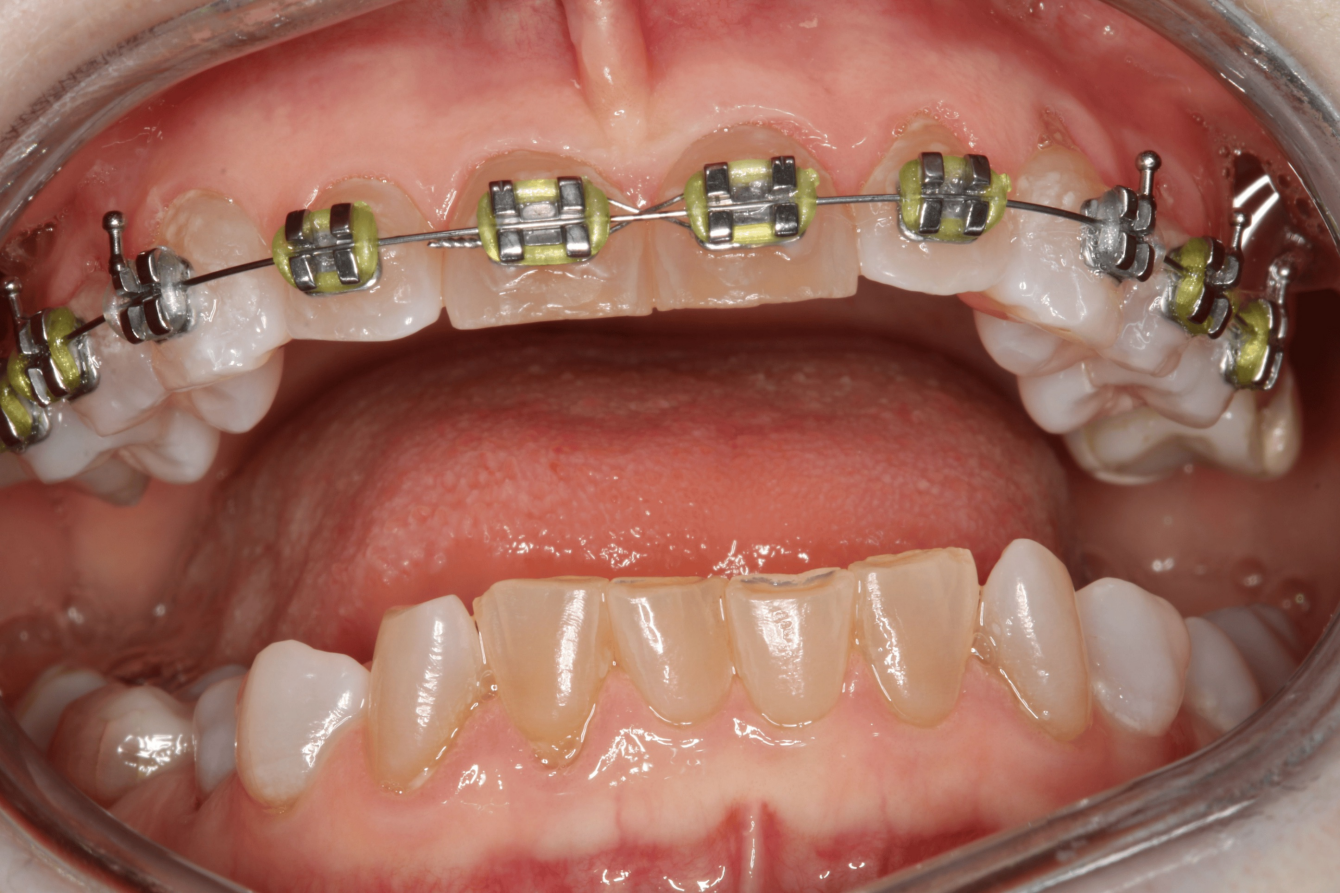
Female patient with dentinogenesis imperfecta

Figure 3 shows a 28-year-old female patient with dentinogenesis imperfecta who is undergoing orthodontic treatment.

Definition and Classification

Dentinogenesis imperfecta is an autosomal dominant inherited disorder with a prevalence of one in 6,000 to one in 8,000 [6,7]. It is divided into three types [65]. Type I is associated with osteogenesis imperfecta. Type II is an isolated form with no systemic disorders. Type III is extremely rare, occurring only within the Brandywine population in Maryland.

De La Dure-Molla et al. emphasise the importance of accurate diagnosis for appropriate treatment and recommend genetic testing to determine the underlying gene mutations [7].

Clinical Criteria

Patients present with opaque, yellowish, and amber teeth with a high rate of chipping. Due to pulp obliteration and short root structures, treating these patients presents a significant challenge [66].

Treatments

The five case reports show a similar treatment assessment for *amelogenesis imperfecta *(Table 3). In restorative rehabilitation, composite fillings and steel crowns were commonly used for children [67-70]. Implants and dentures were used for prosthetic treatment to restore the vertical dimension [67,71]. Due to interdisciplinary approaches, a multidisciplinary team of dentists, orthodontists, and dental surgeons was often involved [67-71].

**Table 3:** Case reports of dentinogenesis imperfecta (n = 5)

Author, year	Alrashdi et al., 2020 [67]		Kaur et al., 2021 [68]		Bouvier et al., 2008 [69]		Garces et al., 2018 [70]	Bencharit et al., 2014 [71]
Country	Saudi Arabia		India		France		USA	USA
Diagnosis	Type II	Type II	Type II		-		Type II	Type II
Relatives also affected	Mother, siblings		-	-	Aunt		No	Father, siblings
Age in years	5	7	7	8	6	14 1/2	12	33
Gender	f	m	m	m	f		m	f
Findings	Loss of the vertical dimension, attrition, prognathism	Poor oral hygiene, lack of compliance, decayed teeth, attrition	Aesthetic impairment, impacted and missing teeth, delayed eruption, loss of chewing function, caries lesion, mixed dentition, bulbous crowns, short root, large pulp chamber	Pain, caries lesion on the molars with abscess, missing strip crowns	Aesthetic impairment, excessive attrition of deciduous teeth, loss of vertical occlusal dimension, obliterated canals, bulbous crowns		Aesthetic impairment, attrition, caries lesions, gingivitis, obliterated canals, bulbous crowns	Soft teeth that break easily, repeated renewal of the fillings, tooth loss due to traumatic chewing contact, temporomandibular joint problems, insufficient fillings, gingivitis
Treatment	Deciduous molar extraction, overlay denture, bite elevation, histologic analysis, paediatric prosthesis	Anaesthesia, extractions, steel crown restoration, glass ionomer cement filling in the front	Extraction, steel crowns on deciduous molars and permanent molars, space maintainers in denture form, strip crowns on incisors in the upper and lower jaw, did not come back to restore molars	Modification of the prosthesis to ensure breakthrough, apexification, lab-fabricated acrylic crowns in the front	Initial phase: steel crowns on deciduous molars and 6 polycarbonate crowns on incisors. Plastic crowns on canines and premolars after eruption and steel crowns on molars after eruption	Secondary phase: removal of temporary crowns, preparation, model fabrication, porcelain-fused-to-metal crowns	Oral hygiene instructions, fluoride application, composite filling, model fabrication, denture fabrication over permanent teeth	Scaling and root planing, oral hygiene instructions, bite elevation with splint of 2 mm in the posterior region, diagnostic wax-up, extraction, 4 implants, preparation of remaining teeth, lithium disilicate crowns, splint therapy
Follow-up	-	-	3 months	3 months	Every 3 months for 1 year		1 week, 1 month, 6 months	6 months
Other	-	autism	-		-		-	-

A summary of the case reports and studies concerning amelogenesis imperfecta and dentinogenesis imperfecta is provided in Table 4.

**Table 4:** Evaluation of the case reports and studies

	Amelogenesis imperfecta case reports (n = 48)		Amelogenesis imperfecta study reports (n = 2)	Dentinogenesis imperfecta case reports (n = 5)	
	Number	No specification	Number	Number	No specification
Patient	n = 55		n = 23	n = 6	
female	n = 31	n = 1	n = 14	n = 3	
male	n = 23		n = 9	n = 3	
Age (median)	3 - 48 years (median = 18)	n = 1	8 – 37 years (median = N/A)	5 – 33 years (median = 7.5)	
Amelogenesis imperfecta Type I	n = 34	n = 8	n = 14	-	-
Amelogenesis imperfecta Type II	n = 3		n = 2	-	-
Amelogenesis imperfecta Type III	n = 10		n= 7	-	-
Amelogenesis imperfecta Type IV	-		-	-	-
Dentinogenesis imperfecta Type II	-	-	-	n = 5	n = 1
Relatives also affected	n = 29	n = 17	-	n = 4	n = 1

Discussion

The treatment results show that an interdisciplinary approach and an individual treatment plan are essential for the treatment of amelogenesis imperfecta or dentinogenesis imperfecta. The choice of materials and methods varies from country to country and within the limits of financial resources.

The challenges are that data describing long-term positive outcomes are lacking, there is no standardised treatment scheme, and high-quality materials are not widely available [9,72].

Early Diagnosis and Intervention

Early diagnosis of amelogenesis imperfecta and dentinogenesis imperfecta is essential, as it is the basis for successful treatment and damage prevention. Genetic testing with clinical and radiological assessment should be used to determine the specific form of the disease. Wright JT et al. emphasise the importance of genetic and molecular diagnosis of the disease to provide a personalised treatment approach [3]. The implementation of preventive measures and recalls could slow the progression of the disease. Early intervention, especially in children, may prevent functional and aesthetic disease [9].

Interdisciplinary Treatment Approaches

The treatment of amelogenesis imperfecta and dentinogenesis imperfecta increasingly requires cooperation between the different disciplines of dental medicine. A team of endodontists, orthodontists, and prosthodontists is required to achieve a successful treatment outcome. In a case report by Doruk et al., a combination of orthodontic and orthognathic surgery was used alongside prosthetic treatment to achieve full functional rehabilitation. [28]. In cases of severe malocclusion, prosthodontic or dental surgery may be used to restore the vertical dimension.

Choice of Material

The choice of material plays an important role in the long-term success of the treatment. High-performance materials such as zirconium oxide or e.max ceramic offer excellent aesthetic results and have a high resistance to wear, but are expensive. Steel crowns and fillings are often used in children because they are less expensive and easier to use. One challenge is the longevity of the materials. As the study by Lindunger and Smedberg [63] shows, teeth indirectly restored with ceramics have better results in terms of longevity than composite fillings, which are susceptible to fracture and discolouration.

Psychosocial and Aesthetic Outcomes

Aesthetic limitations due to amelogenesis imperfecta or dentinogenesis imperfecta can often lead to psychosocial distress, more commonly in adolescents. This population reports shame, negative self-esteem, and social withdrawal. Because of this problem, aesthetic rehabilitation should not only aim to restore dental function but also consider the psychosocial aspect.

Alazmah et al. emphasise that early aesthetic treatment increases the self-esteem of children and adolescents [20]. Minimally invasive techniques, such as composite veneers or fillings, could provide adolescents with a temporary improvement while they await prosthetic treatment. 

Long-term Studies and Standardised Protocols

A key problem is the lack of long-term data on the stability of different treatment regimens [9,72]. Many studies and case reports summarize short-term outcomes. Standardised protocols can improve the quality of treatment and provide a standardised approach. Tuponay et al. report that the American Academy of Pediatric Dentistry (AAPD) has drawn up recommendations for the treatment of Amelogenesis imperfecta. However, these are mainly suitable for children [73]. It would be advisable to issue an international recommendation for both diseases.

Cultural and Economic Differences

The availability of modern techniques and materials varies greatly from country to country. While the use of ceramic restorations and implants is common in Western countries, in countries with limited resources, low-budget items such as steel crowns or fillings are used. This makes a difference in the quality of treatment and longevity of results.

## Conclusions

Amelogenesis imperfecta and dentinogenesis imperfecta are complex diseases that require a multidisciplinary and individualised approach. The results of this review article show that restorative and surgical treatments have positive outcomes, although long-term studies and standardised protocols are lacking. Early diagnosis and treatment are essential to minimise the aesthetic, functional, and psychosocial impact. 

The small number of clinical trials and the reliance on case reports limit the validity of the results. A consistent treatment plan could be supported by the development of standardised guidelines. The literature also points out that genetic analyses need to be more closely integrated into the diagnostic process. This could help in the creation of treatment plans and optimise them for each patient.
